# Comment on ‘Dopamine D_2/3_R availability after discontinuation of antipsychotic treatment: a [^11^C]raclopride PET study in remitted first-episode psychosis patients’ by de Beer et al. 2025

**DOI:** 10.1017/S0033291725102213

**Published:** 2025-11-19

**Authors:** Martin Korsbak Madsen

**Affiliations:** 1 Neurobiology Research Unit, Copenhagen University Hospital, Rigshospitalet, Copenhagen, Denmark; 2 Department of Psychiatry Odense-Svendborg, Svendborg, Denmark

**Keywords:** PET, antipsychotics, dopamine, raclopride, occupancy

To the editorial office at Psychological Medicine,

A recently published paper in Psychological Medicine (de Beer et al., 2025) reports that striatal [^11^C]raclopride BP_ND_ was lower in users of aripiprazole (*n* = 6) compared to both D2 antagonist users and healthy controls 1 week after antipsychotic discontinuation and increased (normalized) after 6–8 weeks. The authors claim that ‘the robust differences’ observed ‘are too large to be solely the result of residual aripiprazole’. Although the study aim and effort deserve praise, there is good reason to believe the results are substantially influenced by drug occupancy at the receptor, possibly also by confounding, and the conclusion consequently wrong.

**Figure 1.**
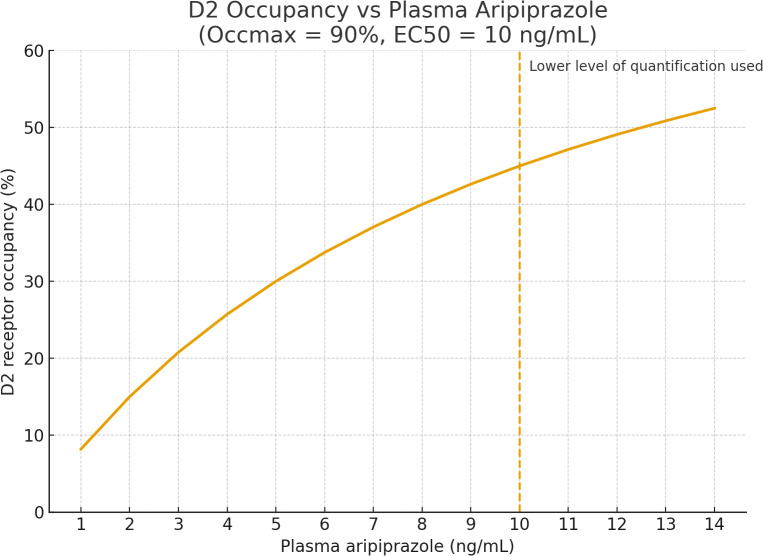
Graph based on data from Gründer et al. (2008). Graph generated using ChatGPT5.

Aripiprazole and its metabolite dehydroaripiprazole have long half-lives (T^1^/_2_ aripiprazole = 75 hours, T^1^/_2_ dehydroariprazole = 94 hours) and very high D2 receptor affinity (Hart, Schmitz, & Gründer, 2022). A one-week time frame from drug discontinuation to PET scan only corresponds to 1.8 and 2.2 half-lives, respectively. Even low plasma levels give rise to substantial receptor occupancy (Figure 1). The authors note that two of the six aripiprazole users had quantifiable levels of aripiprazole (12 and 13 ng/ml) but still include the data in the analysis. Using reasonable empirical values (EC_50_ = 10 ng/L and maximum occupancy = 90%; Gründer et al., 2008), the corresponding receptor occupancy can be estimated to be high: 49 and 51%. This means that true striatal [^11^C] raclopride BP_ND_ for these two observations is really twice as large as what is used in the analysis.

Further, this problem presumably also applies to the BP_ND_ estimates of the remaining four subjects. The paper states that aripiprazole is not detected in the blood for the remaining four subjects at week 1. However, the authors do not state what the lower level of detection is, which is critical for assessing whether drug occupancy is problematic. Plasma concentrations reported for standard therapeutic drug monitoring are commonly not reported below the lower level of quantification. Thus, the information available to the authors was likely only that the plasma levels were below the lower level of quantification (<10 ng/mL). There is thus a need to clarify whether the aripiprazole plasma concentration was below the lower level of quantification or below the lower level of detection and also state what this level was in order to assess to which degree these BP_ND_ estimates are also tainted. A further consideration is also whether dehydroaripiprazole is also measured.

Additionally, confounding may also pose a problem. The study finds that relapse is more common in former antagonist users than in former aripiprazole users. However, there is no information available regarding switching. Many patients may try a partial agonist as one of the first drugs due to a more advantageous side effect profile but switch to a D2 antagonist if there are insufficient antipsychotic effects or there are unacceptable side effects of the partial agonist. Thus, it is possible that the analyses (both relapse and BP_ND_) suffer from confounding. This point is also relevant for the HAMLETT trial’s other publication on this topic, which was recently published in World Psychiatry (Gangadin et al., 2025). Here, the authors reported higher relapse rates in high-affinity antagonist users – but switching information was also not made available and not included in the analyses.

Thus, the analysis of de Beer and colleagues is unfortunately flawed, warranting its reconsideration. It is possible to correct the BP_ND_ estimates in the aripiprazole group by estimating occupancy to the extent useful aripiprazole/dehydroaripiprazole plasma concentration data is available.

Kind regards,

Martin Korsbak Madsen
